# Supplementary material for: A Humanized Yeast Phenomic Model of Deoxycytidine Kinase to Predict Genetic Buffering of Nucleoside Analog Cytotoxicity
Source: Genes (Basel). 2019 Sep 30;10(10):770. doi: 10.3390/genes10100770 (PMC6826991; doi:10.3390/genes10100770)
Supplement: Supplementary file 1 [file genes-10-00770-s001.zip › Additional_Files/Additional_File7_GOTermHeatmaps.zip!/Additional_File7_GOTermHeatmaps/ubiquitin_homeostasis.pdf]

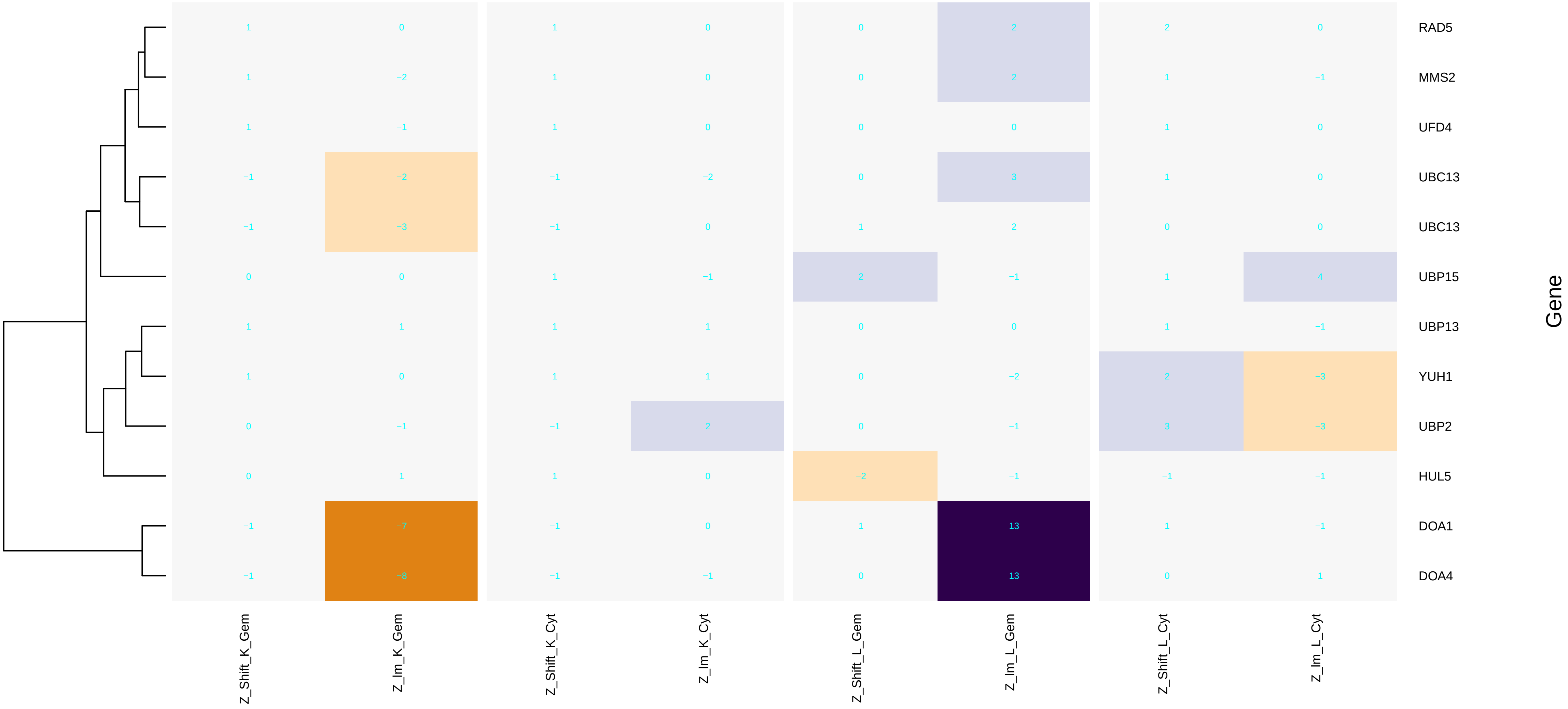

RAD5 1 0 1 0 0 2 2 0
MMS2 1 −2 1 0 0 2 1 −1
UFD4 1 −1 1 0 0 0 1 0
UBC13 −1 −2 −1 −2 0 3 1 0
UBC13 −1 −3 −1 0 1 2 0 0
UBP15 0 0 1 −1 2 −1 1 4
UBP13 1 1 1 1 0 0 1 −1
YUH1 1 0 1 1 0 −2 2 −3
UBP2 0 −1 −1 2 0 −1 3 −3
HUL5 0 1 1 0 −2 −1 −1 −1
DOA1 −1 −7 −1 0 1 13 1 −1
DOA4 −1 −8 −1 −1 0 13 0 1
Gene
Z_Shift_K_Gem
Z_Im_K_Gem
Z_Shift_K_Cyt
Z_Im_K_Cyt
Z_Shift_L_Gem
Z_Im_L_Gem
Z_Shift_L_Cyt
Z_Im_L_Cyt
